# PLCζ Induced Ca^2+^ Oscillations in Mouse Eggs Involve a Positive Feedback Cycle of Ca^2+^ Induced InsP_3_ Formation From Cytoplasmic PIP_2_

**DOI:** 10.3389/fcell.2018.00036

**Published:** 2018-04-03

**Authors:** Jessica R. Sanders, Bethany Ashley, Anna Moon, Thomas E. Woolley, Karl Swann

**Affiliations:** ^1^School of Medicine, Cardiff University, Cardiff, United Kingdom; ^2^School of Biosciences, Cardiff University, Cardiff, United Kingdom; ^3^School of Mathematics, Cardiff University, Cardiff, United Kingdom

**Keywords:** Ca^2+^ oscillations, phospholipase C, strontium, inositol trisphosphate, egg, phosphatidyl inositol bisphosphate

## Abstract

Egg activation at fertilization in mammalian eggs is caused by a series of transient increases in the cytosolic free Ca^2+^ concentration, referred to as Ca^2+^ oscillations. It is widely accepted that these Ca^2+^ oscillations are initiated by a sperm derived phospholipase C isoform, PLCζ that hydrolyses its substrate PIP_2_ to produce the Ca^2+^ releasing messenger InsP_3_. However, it is not clear whether PLCζ induced InsP_3_ formation is periodic or monotonic, and whether the PIP_2_ source for generating InsP_3_ from PLCζ is in the plasma membrane or the cytoplasm. In this study we have uncaged InsP_3_ at different points of the Ca^2+^ oscillation cycle to show that PLCζ causes Ca^2+^ oscillations by a mechanism which requires Ca^2+^ induced InsP_3_ formation. In contrast, incubation in Sr^2+^ media, which also induces Ca^2+^ oscillations in mouse eggs, sensitizes InsP_3_-induced Ca^2+^ release. We also show that the cytosolic level Ca^2+^ is a key factor in setting the frequency of Ca^2+^ oscillations since low concentrations of the Ca^2+^ pump inhibitor, thapsigargin, accelerates the frequency of PLCζ induced Ca^2+^ oscillations in eggs, even in Ca^2+^ free media. Given that Ca^2+^ induced InsP_3_ formation causes a rapid wave during each Ca^2+^ rise, we use a mathematical model to show that InsP_3_ generation, and hence PLCζ's substate PIP_2_, has to be finely distributed throughout the egg cytoplasm. Evidence for PIP_2_ distribution in vesicles throughout the egg cytoplasm is provided with a rhodamine-peptide probe, PBP10. The apparent level of PIP_2_ in such vesicles could be reduced by incubating eggs in the drug propranolol which also reversibly inhibited PLCζ induced, but not Sr^2+^ induced, Ca^2+^ oscillations. These data suggest that the cytosolic Ca^2+^ level, rather than Ca^2+^ store content, is a key variable in setting the pace of PLCζ induced Ca^2+^ oscillations in eggs, and they imply that InsP_3_ oscillates in synchrony with Ca^2+^ oscillations. Furthermore, they support the hypothesis that PLCζ and sperm induced Ca^2+^ oscillations in eggs requires the hydrolysis of PIP_2_ from finely spaced cytoplasmic vesicles.

## Introduction

The fertilization of a mammalian egg involves a series of low frequency Ca^2+^ oscillations that last for many hours. Such Ca^2+^ oscillations play the key role in egg activation and the subsequent development of the embryo (Stricker, 1999). The first Ca^2+^ increase takes ~10 s to travel as a wave across the egg from the point of sperm entry (Miyazaki et al., 1986; Deguchi et al., 2000). However, all the subsequent Ca^2+^ transients have a rising phase of about 1 s which is due to a fast Ca^2+^ wave (>50 μm/s) that crosses the egg from apparently random points in the egg cortex (Deguchi et al., 2000). Each Ca^2+^ increase during the oscillations is due to release from internal Ca^2+^ stores via inositol 1,4,5-trisphophate receptors (IP3R) which are exclusively of type 1 IP3R in mammalian eggs (Miyazaki, 1988; Miyazaki et al., 1993). The sperm stimulates the Ca^2+^ oscillations via inositol 1,4,5-trisphosphate (InsP_3_) production, and all the reproducible studies suggest that this is principally due to the introduction of a sperm specific phospholipase Czeta (PLCζ) into the egg after gamete fusion (Saunders et al., 2002). Injection of PLCζ protein or cRNA causes prolonged Ca^2+^ oscillations that mimic those seen at fertilization in eggs of mice, rat, humans, cows, and pigs (Cox et al., 2002; Saunders et al., 2002; Fujimoto et al., 2004; Kouchi et al., 2004; Kurokawa et al., 2005; Bedford-Guaus et al., 2008; Ito et al., 2008; Ross et al., 2008; Yoon et al., 2012; Sato et al., 2013). PLCζ is distinctive compared to most mammalian PLC isozymes in that it is stimulated by low levels of Ca^2+^ such that it is maximally sensitive to Ca^2+^ around the resting levels in eggs (Nomikos et al., 2005). PLCζ is expected to diffuse across the egg in about 10 min following sperm-egg fusion, hence the fast Ca^2+^ waves seen after the initial Ca^2+^ transient are propagated within a cytoplasm in which PLCζ has probably dispersed throughout the egg.

There are two classes of model to explain how InsP_3_ causes Ca^2+^ oscillations in cells, both which have been proposed for fertilizing mammalian eggs (Dupont and Goldbeter, 1994; Politi et al., 2006). There are some models that propose Ca^2+^ dependent sensitization, and then de-sensitization, of the IP3R is necessary to generate each Ca^2+^ transient (Politi et al., 2006). This class of models supports the finding that mouse and hamster eggs can be stimulated to oscillate by sustained injection of InsP_3_, or by injection of the IP3R agonist adenophostin (Swann et al., 1989; Brind et al., 2000; Jones and Nixon, 2000). On the other hand there are other models in which Ca^2+^ dependent production of InsP_3_ generates each Ca^2+^ transient, and in which InsP_3_ is predicted to oscillate alongside Ca^2+^ (Politi et al., 2006). This second class of model is supported by the detection of InsP_3_ oscillations in mouse eggs injected with PLCζ, albeit at high levels of PLCζ (Shirakawa et al., 2006). However, it is not clear if any oscillatory changes in InsP_3_ oscillations are necessary for generating Ca^2+^ increases. Either classes of model have to incorporate the observation that the Ca^2+^ oscillations have a dependence upon Ca^2+^ influx. So for example, if fertilizing hamster or mouse eggs are incubated in Ca^2+^ free media the oscillations run down and stop (Igusa and Miyazaki, 1983; Lawrence and Cuthbertson, 1995; McGuinness et al., 1996). It has been suggested that the Ca^2+^ store content is critical in setting the timing of Ca^2+^ oscillations in mouse eggs. This is supported by evidence that the SERCA inhibitor thapsigargin can also be used to block sperm and PLCζ induced Ca^2+^ oscillations by depleting Ca^2+^ stores content (Kline and Kline, 1992b). However, changes in cytosolic Ca^2+^ may also play a role in the timing of oscillations since cytosolic Ca^2+^ can regulate both IP3Rs and PLCζ activity.

Sustained Ca^2+^ oscillations in mouse eggs can also be triggered by incubation in media containing Sr^2+^ instead of Ca^2+^ (Kline and Kline, 1992a; Bos-Mikich et al., 1995). Sr^2+^-induced Ca^2+^ oscillations resemble those seen at fertilization, and they are as effective as fertilization or PLCζ in triggering development to the blastocyst stage (Yu et al., 2008). The oscillations are dependent upon Sr^2+^ influx into the egg and the presence of functional IP3Rs (Zhang et al., 2005). However, it is not clear how Sr^2+^ causes Ca^2+^ oscillations. One study suggested that the effect of Sr^2+^ requires InsP_3_ production (Zhang et al., 2005). However, unlike fertilization, there is no Sr^2+^ induced downregulation of IP3Rs and this suggests that Sr^2+^ does not cause any substantial InsP_3_ generation (Jellerette et al., 2000). *In vitro* preparations of IP3Rs receptors can be stimulated to open by Sr^2+^ ions (Marshall and Taylor, 1994), so a direct effect of Sr^2+^ on IP3Rs is also likely, but any changes in InP_3_ sensitivity in eggs have yet to be shown.

As well as its high sensitivity to Ca^2+^, another unusual characteristic of PLCζ is that it does not localize to the plasma membrane (Yu et al., 2012). The substrate for PLCζ, phosphatidylinositol 4,5-bisphophate (PIP_2_), can be detected in the plasma membrane of mouse eggs using the PH domain of PLCδ1 (Halet et al., 2002), but the depletion of such PIP_2_ from the plasma membrane does not affect the generation of Ca^2+^ oscillations in response to PLCζ or fertilization (Yu et al., 2012). In contrast to somatic cells, mouse eggs have been shown to contain PIP_2_ in intracellular vesicles (Yu et al., 2012). These vesicles were detected using PIP_2_ antibodies and were found to be dispersed throughout the cytoplasm of mouse eggs (Yu et al., 2012). PLCζ can also be detected on small cytoplasmic vesicles using immunostaining (Yu et al., 2012). The significance of this type of intracellular localization of PLCζ and PIP_2_ has not been made clear.

Here we report experiments that analyse the mechanism of PLCζ induced Ca^2+^ oscillations in mouse eggs. We use photo-release of caged InsP_3_ to show that PLCζ causes Ca^2+^ oscillations via a positive feedback cycle of Ca^2+^ release and Ca^2+^ induced InsP_3_ production. In contrast the Sr^2+^ induced Ca^2+^ oscillations in mouse eggs involve a sensitization of InsP_3_ induced Ca^2+^ release. We go on to show that the cytosolic Ca^2+^ is more likely to be important for setting the pace of oscillations in eggs than Ca^2+^ store content. In addition, we present simulations to show that the restricted diffusion of InsP_3_ in cytoplasm implies that the source of InsP_3_ generation, PIP_2_, needs to be dispersed through the egg interior to account for PLCζ induced rapid Ca^2+^ waves. Finally, we provide further evidence that PIP_2_ is present on intracellular vesicles in eggs and that this is required for PLCζ and sperm induced Ca^2+^ oscillations in eggs.

## Materials and methods

### Handling and microinjection of mouse Eggs

MF1 mice between 6 and 8 weeks of age were injected with pregnant mare's serum gonadotrophin (PMSG, Intervet) followed by human chorionic gonadotrophin (hCG, Invervet) ~50 h later (Fowler and Edwards, 1957). Eggs were collected from these mice 15 h after HCG injection, from the dissected ovaries. All animal handling and procedures were carried out under a UK Home Office License and approved by the Animal Ethics Committee at Cardiff University. Once collected, the eggs were kept at 37°C in M2 media (Sigma Aldrich). All Ca^2+^ dyes and intracellular probes were introduced into the cytosol of the eggs using a high pressure microinjection system with the eggs maintained in M2 media throughout (Swann, 2013). For *in vitro* fertilization sperm was collected from the epididymis of F1 C57/CBA hybrid male mice. The sperm were isolated in T6 media containing 16 mg/ml bovine serum albumin (BSA, Sigma Aldrich) and left to capacitate for 2–3 h before adding to eggs (Yu et al., 2012).

### Measurements and analysis of intracellular Ca^2+^ and InsP_3_ uncaging

In all experiments cytosolic Ca^2+^ was measured using fluorescent Ca^2+^ indictor Oregon Green BAPTA dextran (OGBD) (Life Technologies). OGBD was diluted in a KCl HEPES buffer (120 mM KCl, 20 mM HEPES at pH 7.4) so that the injection solution contained 0.33 or 0.5 mM OGBD. The OGBD mix was microinjected into eggs using high pressure pulses. In those eggs that were stimulated by adenophostin this was microinjected into eggs along with the OGBD. In this case instead of mixing the OGBD with KCL HEPES it was mixed with KCL HEPES containing 5 μM adenophostin in the same quantities. Where PLCζ cRNA was used this was microinjected alongside OGBD in the same way at a concentration of 0.02 μg/μl. For imaging, eggs were then transferred to a glass-bottomed dish, containing HKSOM media, on an epifluorescence imaging system (Nikon TiU) attached to a cooled CCD camera as described previously (Swann, 2013). Ca^2+^ dynamics were measured using the time-lapse imaging mode of Micromanager software (https://micro-manager.org/) where an image was captured every 10 s. Where IVF was performed, or drugs were later added to the eggs, the zona pellucidas were removed from the eggs using acid Tyrodes treatment prior to imaging. For those experiments that required InsP_3_ stimulation, NPE-caged-InsP_3_ (1 mM in the pipette) from ThermoFisher Scienific was microinjected prior to imaging at the same time as the injection of fluorescent dye (OGBD). In order to photo-release InsP_3_ the eggs were exposed to an electronically gated UV LED light source (365 nm, Optoled Lite, Cairn Research Ltd) that was positioned just above the dish containing the eggs. The duration of the UV pulse was controlled by a time gated TTL pulse that was delivered in between two successive fluorescence acquisitions. All data measuring Ca^2+^ dynamics were recorded as .tif files using the Micromanger software on the epifluorescence system.

#### Media, chemicals, and drugs

M2 media was purchased from Sigma Aldrich as a working solution. HKSOM was made up to pH 7.4, in cell culture grade water as follows: 95 mM NaCl, 0.35 mM KH2PO4, 0.2 MgSO4, 2.5 mM KCl, 4 mM NaHCO_3_, 20 mM HEPES, 0.01 mM EDTA, 0.2 mM Na Pyruvate, 1 mM L-glutamine, 0.2 mM glucose, 10 mM Na Lactate 1.7 mM CaCl_2_, 0.063 g/l Benzylpenicllin, and 10 mg/l phenol red. Ca^2+^ free media was made in the same way as HKSOM however CaCl_2_ was not added and the media was supplemented with 100 μM EGTA. Sr^2+^ containing media was made in the same way as HKSOM however, instead of adding 1.7 mM CaCl_2_, 10 mM SrCl_2_ was added instead.

All drugs and chemicals used, unless otherwise mentioned, were purchased from Sigma Aldrich. Propranolol was used at a working concentration of 300 μM in HKSOM media. A stock of 300 mM propranolol was made up in DMSO which was then diluted 1:100 in HKSOM media. Then 100 μl of this solution was pipetted into the imaging dish containing 900 μl of standard HKSOM. Propranolol was removed by washing out this media and replacing it with fresh HKSOM media using a perfusion system that passed 10 ml of clean HKSOM through the dish containing the eggs to ensure sufficient wash out. In a similar way a stock of 5 mM thapsigarin in DMSO was diluted 1:1,000 to a concentration of 5 μM in HKSOM and then 100 μl of this thapsigargin solution was added to the imaging dish containing 900 μl of HKSOM to give a working concentration of 500 nM of thapsigargin.

### Confocal imaging

In those eggs that were microinjected with PBP10, a solution of 1 mM PBP10 (Tocris Biosciences, UK) was made up in KCl HEPES and ~4–10 pl of this solution was microinjected into each egg. Following PBP10 microinjection, eggs were imaged on a Leica SP2 Confocal (Leica, Wetzler, Germany) microscope using a Helium-Neon laser (543 nm) at 30% intensity. Eggs were imaged in M2 media using a x63 oil objective and a pinhole aperture of 91 nm. Images were acquired with a line averaging of 8 and a resolution of 1,058 × 1,058 pixels. For each egg a single z-stack image of (1 μm depth) was captured of an equatorial slice through the egg. All images were exported as .tif files and analyzed using Image J (https://imagej.nih.gov/ij/).

### Data analysis

Quantitative data measuring the Ca^2+^ dynamics of the eggs on the widefield imaging system was extracted from .tif stacks using Image J (https://imagej.nih.gov/ij/). Background fluorescence was first subtracted from the egg fluorescence value. These fluorescence values were then normalized by dividing each fluorescence value in the egg by the baseline fluorescence value at the start of the imaging run to provide a relative change in fluorescence (F/F0) that could be plotted against time. These traces were produced and analyzed using SigmaPlot 12. The Confocal images were also analyzed using Image J software. PIP_2_ positive vesicle size and distribution was calculated using the particle analysis function on Image J and a nearest neighbor distance (Nnd) plugin in Image J. A bandpass filter function was applied to the images (large objects were filtered down to 40 pixels and small ones enlarged to 3 pixels). The threshold was altered to between 2 and 5% so only the fluorescence of the vesicles inside the image of the egg were included in the analysis. The particle analysis function was applied and configured so it recorded area, integrated intensity and coordinates for each fluorescent vesicle in the egg. These areas were used to work out the radius and diameter of the vesicles. The coordinates were fed into a nearest distance neighbor plugin (https://icme.hpc.msstate.edu/mediawiki/index.php/Nearest_Neighbor_Distances_Calculation_with_ImageJ) to give the mean distance between the vesicles. The total fluorescence of the vesicles was calculated by adding all the integrated intensity readings for a single egg which was carried out using the measure tool in ImageJ and background fluorescence values were subtracted. Statistical analysis was carried out using SigmaPlot 12. If not stated otherwise the data is presented as the mean and standard errors of the mean. Shapiro–Wilk tests for normality and tests for equal variances were conducted prior to carrying out group comparison tests. If the data passed both these tests a Student's *T-test* was conducted. If the data failed either or both of these tests a Mann-Whitney *U-test* was conducted instead.

### Mathematical method of Ca^2+^ waves

The model and associated parameter values are based on the work of (Politi et al., 2006; Theodoridou et al., 2013). The reaction-diffusion equations define the interactions between free cytosolic calcium, *u*; stored calcium, *v*; and IP3, *p*,

(1) $$\frac{\text{d}u}{\text{d}t}=d\nabla^{2}u+A-D\frac{u^{ed}}{u^{ed}+u_{d}^{ed}}\left(1-\frac{p^{es}}{p^{es}+p_{s}^{es}}\right)+K(u,v,p),$$

(2) $$\frac{\text{d}v}{\text{d}t}=d\nabla^{2}v-K(u,v,p)S(x,y,L_{0}),$$

(3) $$\frac{\text{d}p}{\text{d}t}=d\nabla^{2}p+\epsilon +PLC\frac{u^{ep}}{u^{ep}+u_{p}^{ep}}S(x,y,L)-rp,$$

(4) $$\begin{array}{l} K(u,v,p)=-B\frac{u^{eb}}{u^{eb}+u_{b}^{eb}}\\ \;+C\frac{v^{ec}}{v^{ec}+v_{c}^{ec}}\frac{p^{epc}}{p^{epc}+p_{c}^{epc}}\frac{u^{epa}}{u^{epa}+u_{pa}^{epa}}\left(1-\frac{u^{epi}}{u^{epi}+u_{pi}^{epi}}\right)-Ev. \end{array}$$

(5) $$S(x,y,L)=\{\begin{array}{l} 1\text{ if }\left(\frac{x}{L}-\left\lfloor \frac{x}{L}\right\rfloor \right)<\frac{L_{on}}{L}\text{ and }\left(\frac{y}{L}-\left\lfloor \frac{y}{L}\right\rfloor \right)<\frac{L_{on}}{L},\\ 0\text{ Otherwise}. \end{array}$$

The equations represent interactions in which free Ca^2+^ acts as a self-inhibitor but, along with InsP_3_ and stored Ca^2+^, stimulates the release of stored Ca^2+^, creating a system that can produce oscillations in the concentrations of calcium and InsP_3_. Critically, all species are able to diffuse with the same diffusion coefficient, *d*.

The actions of the stored Ca^2+^ and the InsP_3_ only occur in discrete regions. This spatial discreteness is controlled by the repeating function *S*(*x, y, L*). Essentially, the function *S*(*x, y, L*) creates a regular grid of squares of size *L*_*on*_ × *L*_*on*_ in which the specified kinetics are active. We are then able to alter the wavelength, or separation distance, *L*, between these active regions.

The equations were simulated using a finite element Runge-Kutta method on a two-dimensional disk of diameter 70 μm, which was discretised into 6,550 elements. The 2D assumption is considered valid because any dilution effects of going to three dimensions are off set equally by an increase in the third dimension production. The two-dimensional simulations can be thought of a single slice through a cell and it offers speed, clarity and insight. Finally, the boundary was specified to have a zero-flux condition, meaning that no substances were able to leak out of the domain. This is a simplification considered valid since it is known that PLCζ induced Ca^2+^ spikes can be generated in mouse eggs where no membrane Ca^2+^ fluxes occur (Miao et al., 2012). The equations are accompanied by the parameter values specified in Table 1, where all unit dimensions are chosen to make *u*, *v*, and *p* have units of μMol, space is in μm and time is in seconds. The initial conditions for all populations were at steady state except for a small perturbation of a two-dimensional Gaussian profile at the point (20,20), in the free Ca^2+^ population.

**Table 1:** Parameter values for Equations (1)–(5).

**Parameter**	**Value**	**Definition**
*A*	0.25	Calcium source
*B*	200	Strength of calcium induced calcium degradation
*C*	3, 125	Calcium release depending on all forms of calcium and IP_3_
*D*	7.5	Strength of IP_3_ blocking calcium degradation
*E*	0.00125	Calcium leakage
*PLC*	100	Strength of calcium induced IP_3_ release
ϵ	0.001	IP_3_ source
*r*	10	IP_3_ degradation
*d*	10	Diffusion rate
*u*_*d*_	0.5	Calcium degradation sensitivity to calcium
*ed*	2	Hill coefficient
*p*_*s*_	0.1	Calcium degradiation sensitivity to IP_3_
*es*	3	Hill coefficient
*u*_*p*_	0.025	IP_3_ production sensitivity to calcium
*ep*	4	Hill coefficient
*u*_*b*_	2.25	Calcium degradation sensitivity to calcium
*eb*	2	Hill coefficient
*v*_*c*_	9	Calcium release sensitivity to stored calcium
*ec*	2	Hill coefficient
*u*_*pa*_	0.45	Calcium release sensitivity to cytosolic calcium
*epa*	4	Hill coefficient
*u*_*pi*_	1	Calcium release sensitivity to cytosolic calcium
*epi*	5	Hill coefficient
*p*_*c*_	0.1	Calcium release sensitivity to IP_3_
*epc*	2	Hill coefficient
*L*_0_	1.5	Calcium store spacing

*All unit dimensions have been chosen to make u, v, and p have units of μMol, space is in μm and time is in seconds*.

## Results

### PLCζ and Sr^2+^ trigger Ca^2+^ oscillations in eggs via different mechanisms

**Figure 1:**
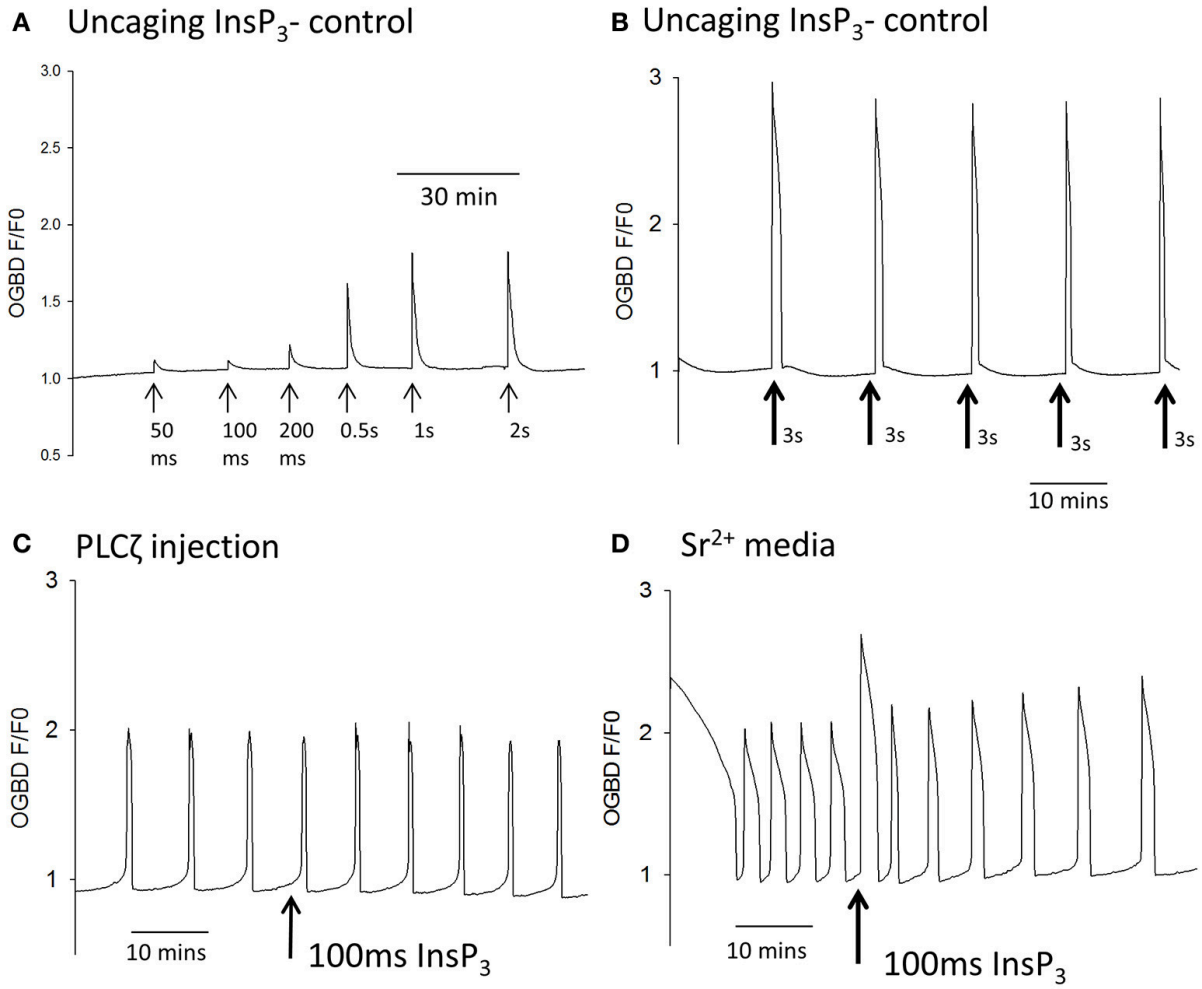
Ca^2+^ oscillations and uncaging pulses of InsP_3_. In **(A)** an example trace is shown of Ca^2+^ increases (as measured by OGBD fluorescence) in an egg in response to different amounts of InsP_3_. Eggs were injected with caged InsP_3_ and exposed to varying durations of UV light pulse (from 50 ms to 2 s) to photo-release the InsP_3_ (trace typical of *n* = 21 eggs). In this and all other traces shown, the pulses were applied at points indicated by the arrows. In **(B)** an example trace is shown of changes in cytosolic Ca^2+^ in an egg in response to the “uncaging” of caged InsP3 using long duration UV pulses of 3 s. Arrows indicate where pulses of UV light were applied (typical of *n* = 7 eggs). In **(C)** an example trace is shown of changes in cytosolic Ca^2+^ in an egg stimulated following the microinjection of mouse derived PLCζ cRNA (0.02 μg/μl) and caged InsP_3_. The arrow indicates where a 100 ms pulse of UV light was applied (*n* = 14 eggs). In all 14/14 such recordings there was no sudden increase in Ca^2+^ even when the pulse was applied during the pacemaker rising phase of Ca^2+^. In **(D)** an example trace is shown with changes in cytosolic Ca^2+^ in an egg stimulated by media containing 10 mM Sr^2+^. The arrow indicates where a 100 ms pulse of UV light was applied to uncage InsP_3_ (*n* = 32 eggs). In all 32/32 cases there was a rapid Ca^2+^ increase that started with the very next OGBD fluorescence measurement after the UV pulse (<10 s).

We investigated the mechanism generating Ca^2+^ oscillations by using photo-release of caged InsP_3_ that was microinjected into mouse eggs. In initial experiments we uncaged InsP_3_ in unfertilized (control) mouse eggs that were not undergoing any Ca^2+^ oscillations. Figure 1A shows that UV pulses of light from 50 ms through to 2 s generated Ca^2+^ increases with the amplitudes that were larger with longer duration pulses. With the protocol we used there was adequate amounts of caged InsP_3_ for multiple releases of InsP_3_ even with longer duration pulses of UV light as illustrated by Figure 1B which shows that 3 s pulses could generate repeated large rises in Ca^2+^ in control eggs. We then tested the effects of triggering such pulses during Ca^2+^ oscillations induced by either Sr^2+^ media or by PLCζ injection. Figure 1C shows that when a 100 ms pulse was used in eggs injected with PLCζ the uncaging of InsP_3_ caused no Ca^2+^ increase. In contrast, Figure 1D shows Ca^2+^ oscillations occurring in response to Sr^2+^ media and in such eggs there was a rapid and large Ca^2+^ transient every time a pulse of just 100 ms was used to uncage InsP_3_. Since the response to 100 ms pulses of UV were minimal in control eggs (Figure 1A) these data show that Sr^2+^ media sensitizes eggs to InsP_3_ induced Ca^2+^ release and that, in contrast, IP3R are not sensitized to InsP_3_ by PLCζ injection.

**Figure 2:**
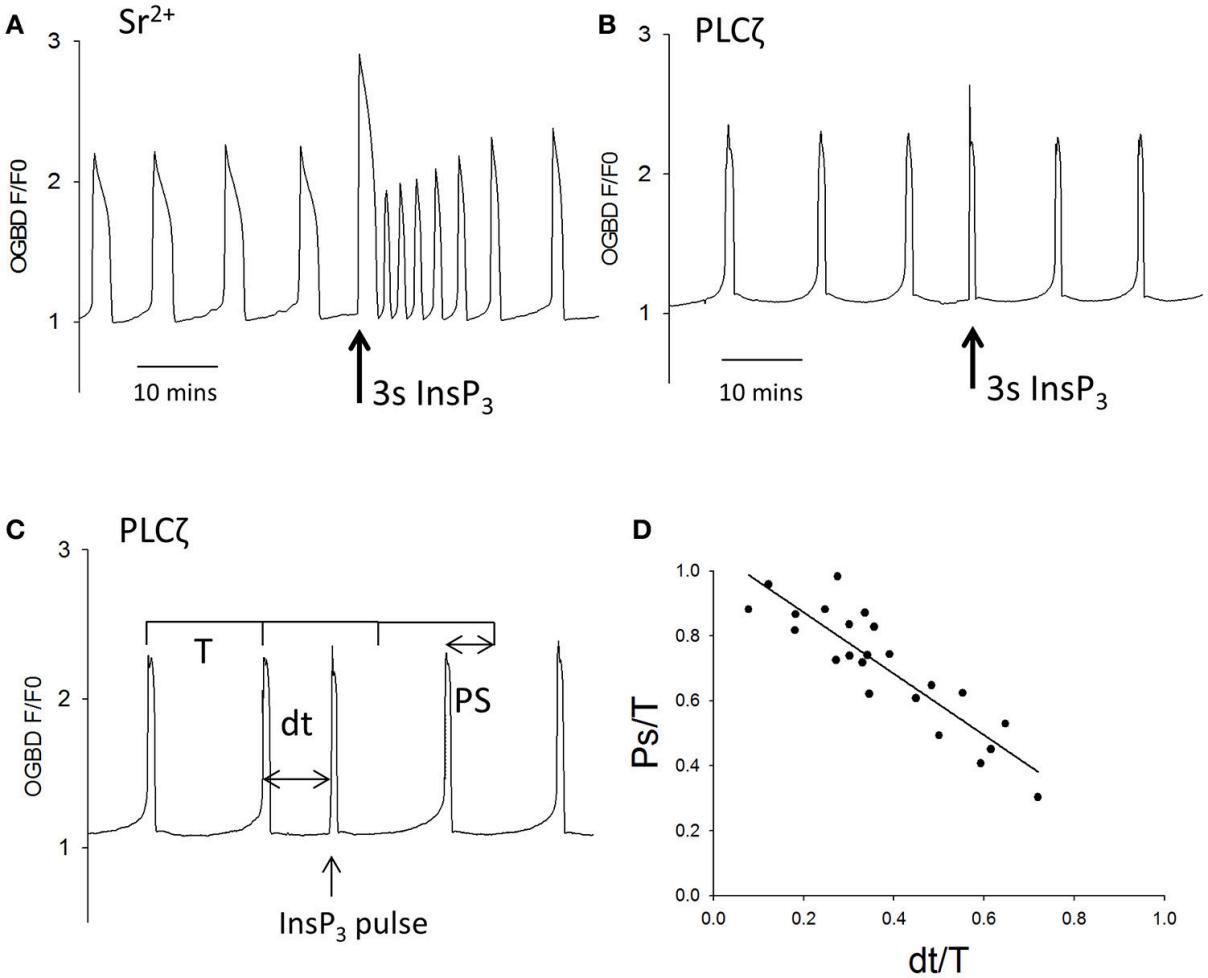
The effect of large pulses of InsP_3_ on PLCζ or Sr^2+^ triggered Ca^2+^ oscillations. **(A)** shows an example of the way eggs responded a large uncaging pulse of InsP_3_ (3 s UV light at the arrow) by an increase in the frequency of Sr^2+^ triggered Ca^2+^ oscillations (*n* = 20 eggs). There was a significant increase in frequency of Ca^2+^ spikes from 3.45 (±0.27 sem) in 20 min to 5.05 (±0.35 sem) in 20 min (*p* < 0.001). In **(B)** a similar experiment is shown but with PLCζ induced Ca^2+^ oscillations. The sample trace in **(B)** shows that a 3 s uncaging pulse of InsP_3_ (at the arrow) caused an immediate Ca^2+^ increase but no increase in frequency (*n* = 23 eggs). We analyzed the ability of such pulses to reset the phase of oscillations by measuring then phase shift (PS) and comparing it to time delay (dt) at which the InsP_3_ pulse was applied. **(C)** Illustrates how these values were measured on an actual sample trace. Each value was divided by the time-period (T) for the oscillations in order to normalize the values between different eggs. **(D)** Shows a plot of these values for all 23 eggs tested.

The two classes of model for Ca^2+^ oscillations, those that involve the dynamic properties of IP3Rs and those that involve InsP_3_ production oscillations, can be distinguished in a definitive manner by examining the response to a sudden pulse of InsP_3_ (Sneyd et al., 2006). Models that are dependent upon IP3R kinetics alone respond to a pulse of InsP_3_ by showing a transient increase in the frequency of Ca^2+^ oscillations (Sneyd et al., 2006). In contrast, models that depend on Ca^2+^ induced InsP_3_ production, and imply InsP_3_ oscillations, respond to a sudden increase in InsP_3_ by showing an interruption of the oscillations which leads to a resetting of the phase of oscillations (Sneyd et al., 2006). We tested the effect of using large uncaging pulses of InsP_3_ on Sr^2+^ induced, or PLCζ induced, Ca^2+^ oscillations in mouse eggs. Figure 2A shows that during Sr^2+^ induced oscillations a 3 s uncaging pulse of InsP_3_ caused a large increase in Ca^2+^ followed by a significant increase in the frequency of Ca^2+^ oscillations. In contrast, with PLCζ induced Ca^2+^ oscillations, Figure 2B shows that the same 3 s uncaging pulse of InsP_3_ did not cause any increase in frequency, but interrupted the periodicity of oscillations leading to a delay before the next Ca^2+^ increase. To confirm that this phenomenon was phase resetting, we plotted the shift in phase (PS) caused by uncaging of InsP_3_ against the time delay (dt) of the InsP_3_ pulse from the subsequent Ca^2+^ spike (see Figure 2C). Each of these values was divided by the time period T in order to take into account the different frequency of Ca^2+^ oscillations in each egg. With phase resetting this plot should give a line from 1 to 1 on each axis, and Figure 2D shows that the data from 23 PLCζ injected eggs exposed to uncaging pulses of InsP_3_ fit closely on such a line. These data clearly show that a pulse of InsP_3_ causes phase resetting of Ca^2+^ oscillations in mouse eggs, which is completely different from that seen with Sr^2+^ induced oscillations. Hence, overall the data suggest that PLCζ and Sr^2+^ media trigger Ca^2+^ oscillations in mouse eggs via fundamentally different mechanisms. Sr^2+^ stimulates IP3Rs to make them effectively more sensitive to InsP_3_, and that PLCζ induced Ca^2+^ oscillations involve Ca^2+^ stimulated InsP_3_ production where InsP_3_ acts as a dynamic variable that should oscillate in synchrony with Ca^2+^ oscillations.

### Cytosolic Ca^2+^ vs. Ca^2+^ stores and the frequency of Ca^2+^ oscillations

**Figure 3:**
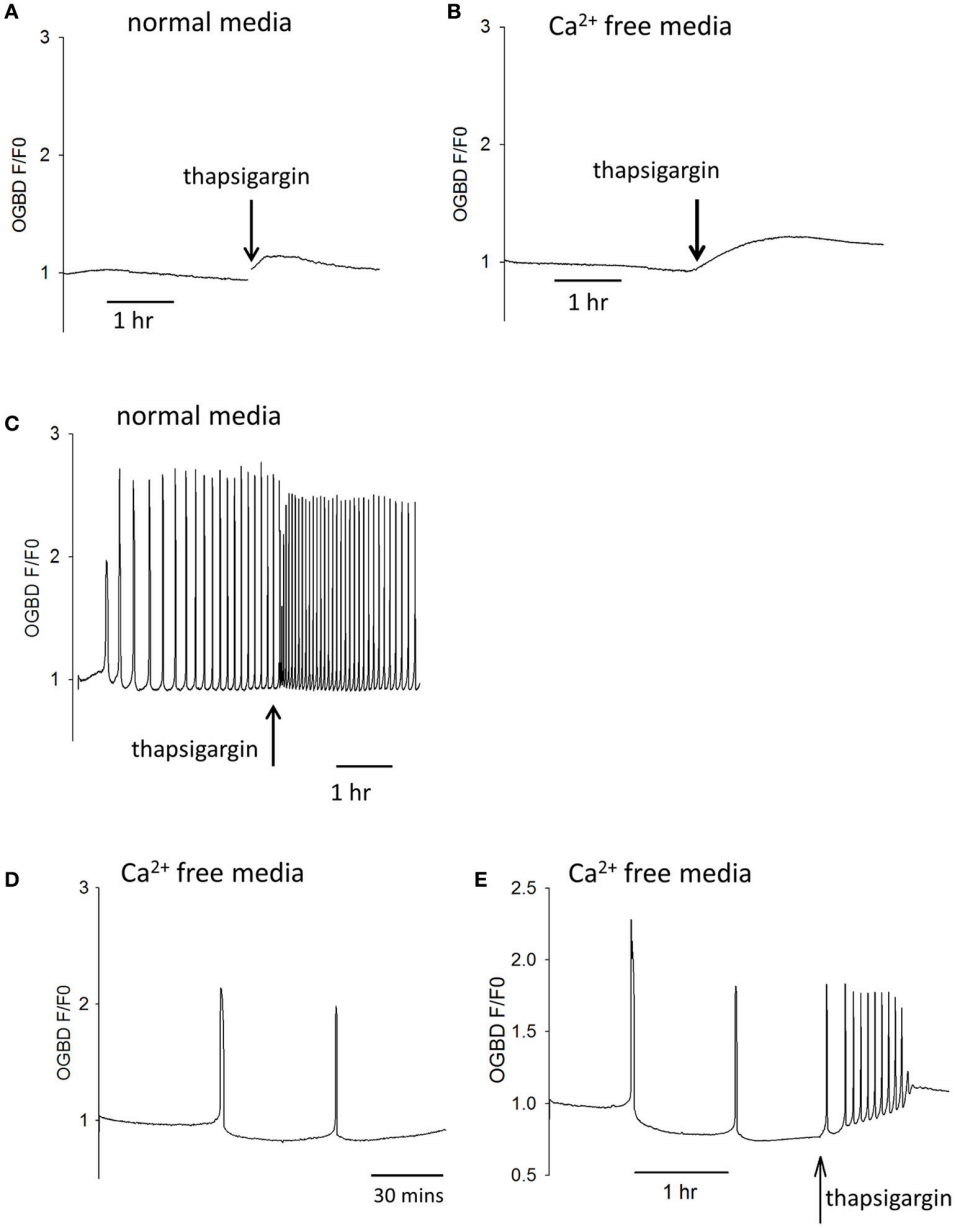
Cytosolic Ca^2+^ and the frequency of Ca^2+^ oscillations. In **(A)** an example is shown of a trace showing changes in cytosolic Ca^2+^ in an egg incubated in normal HKSOM media following the addition of 500 nM thapsigargin (typical of *n* = 12 eggs). The increase in Ca^2+^ was from 0.936 ± 0.013 SEM to 1.07 ± 0.0197 SEM which is a significant (*P* < 0.01). In **(B)** a similar example is shown of the addition 500 nM thapsigargin to an egg in Ca^2+^ free media (containing 100 μM EGTA), which was typical of *n* = 12 eggs. The increase in basal Ca^2+^ was from 0.908 ± 0.0134 SEM up to 1.29 ± 0.0168 SEM which was significant (*P* < 0.001). In **(C)** an example of one of 6 eggs is shown where the same low concentration of thapsigargin increased the frequency of Ca^2+^ oscillations by 1.72-fold (±0.07 SEM). In **(D)** a trace is shown from an egg that was injected with PLCζ RNA and then placed in Ca^2+^ free media. The mean number of Ca^2+^ spikes in such experiments was 1.56 (*n* = 18 eggs, ±0.31 SEM) Ca^2+^ spikes in 10,000 s (2 h 47 min) h. The Ca^2+^ levels decreased to 0.84 ± 0.029 SEM which was significantly less than the starting level (*P* < 0.001). In **(E)** is shown an example of an egg that had been injected with PLCζ RNA and then placed in Ca^2+^ free media as in **(C)**. However, in these experiments 500 nM thapsigarin was added after >2 h. In 16/17 such treated eggs there was an increase in the frequency of Ca^2+^ oscillations. There were an average of 1.77 spikes (±0.18 SEM) before adding thapsigarin but a mean of 7.11 spikes (±1.3 SEM) after thapsigarin addition. The resting Ca^2+^ level increased from 0.84 ± 0.029 SEM, before adding thapsigargin to 1.076 ± 0.017 sem in eggs where it stabilized. This is a signicant increase in Ca^2+^ concentration (*P* < 0.001).

Since Ca^2+^ release and InsP_3_ formation are predicted to form part of a positive feedback loop we decided to re-investigate some observation previously made on Ca^2+^ oscillations in eggs. One finding made in hamster and mouse eggs is that both sperm (and PLCζ)-triggered Ca^2+^ oscillations “run down” and can cease entirely in Ca^2+^ free media (Igusa and Miyazaki, 1983; Lawrence and Cuthbertson, 1995). This phenomena has been explained in terms of Ca^2+^ store depletion but the level of cytosolic Ca^2+^ and its effect on InsP_3_ production could also be important. We re-examined the role of Ca^2+^ stores and resting Ca^2+^ using the SERCA inhibitor thapsigarin. Previous studies used high concentrations (>10 μM) of thapsigargin to completely block Ca^2+^ oscillations in eggs (Kline and Kline, 1992b). To investigate the role of Ca^2+^ store content we used much lower concentrations of thapsigargin which caused only a small elevation of cytosolic Ca^2+^. Figures 3A,B show that the addition of 500 nM thapsigargin to mouse eggs caused a small and prolonged increase in resting cytosolic Ca^2+^ in normal media and Ca^2+^ free media, which is consistent with a slight inhibition of SERCA pumps. When the same concentration of thapsigargin was added to eggs undergoing Ca^2+^ oscillations in response to PLCζ there was a marked acceleration of Ca^2+^ oscillations, and a reduction in the amplitude of Ca^2+^ spikes (Figure 3C). Similar to previous reports, we found that the pattern of PLCζ induced Ca^2+^ oscillations show a run down in Ca^2+^ free media (containing EGTA). We noted that this was associated with a decline in the fluorescence of OGBD, suggesting that resting Ca^2+^ levels were also undergoing a decline (Figure 3D). When low concentrations of thapsigargin (500 nM) were added to PLCζ injected eggs in Ca^2+^ free media there was a restoration of Ca^2+^ oscillations (Figure 3E). It is noteworthy that in Figure 3E the eggs were in Ca^2+^ free media and yet the addition of thapsigargin, which would cause further Ca^2+^ store depletion, actually leads to a restoration of Ca^2+^ oscillations. Nevertheless, the restoration of Ca^2+^ oscillations was associated with a rise in the “basal” Ca^2+^ level (Figure 3E). These data are consistent with the idea that cytosolic Ca^2+^ plays a key role in triggering each Ca^2+^ rise, and that Ca^2+^ stores are not significantly depleted in mouse eggs by incubation in Ca^2+^ free media.

### PLCζ induced Ca^2+^ oscillations and intracellular PIP_2_

**Figure 4:**
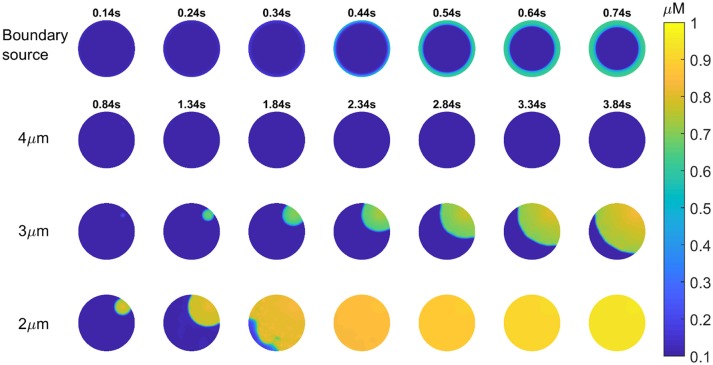
Simulation of InsP_3_ induced Ca^2+^ release in eggs. Images are shown for a 2-dimensional simulation of the propagation of a Ca^2+^ wave in a mouse egg using a mechanism based upon Ca^2+^ induced InsP_3_ formation. Images for each time series is shown in each of the rows. For the first row the only source of PIP_2_ for making InsP_3_ is at the boundary (the plasma membrane) and this does not cause a wave at all. The times for each image (in seconds) in the top row is indicated by numbers above each image. In the next three rows the source of Ca^2+^ induced InsP_3_ formation is spaced at different distances. The time intervals for each image is indicated in the second row and it is then the same for each image going down in each column. In the first row the distance for the PIP_2_ is 4 μm, and again no Ca^2+^ wave can be generated. With a PIP_2_ source spaced at 3 or 2 μm we found that a Ca^2+^ wave can be generated. With a 2 μm separation a wave occurs that crosses the “egg” in ~1 s.

Previous studies of fertilizing mouse and hamster eggs show that most Ca^2+^ waves cross the egg in about 1 s, and propagate through the cytoplasm at speeds in excess of 50 μm/s. This matches the rising phase of (all but the initial) Ca^2+^ transients in mouse eggs which is ~1 s after fertilization or after PLCζ protein injection (Deguchi et al., 2000). Since data in Figure 2 implies that the upstroke of each Ca^2+^ rise involves an InsP_3_ and Ca^2+^ positive feedback loop, then it is necessary for both molecules to be sufficiently diffusible. The Ca^2+^ stores (the endoplasmic reticulum) are spread across the egg. However, this may not be the case with PIP_2_ that is the precursor to InsP_3_. In most cells PIP_2_ is in the plasma membrane, and if this is used in Ca^2+^ waves in eggs then InsP_3_ diffusion range might constrain the ability to generate fast Ca^2+^ waves. Recently, the diffusion coefficient of InsP_3_ in intact cells has been shown to be <10 μm^2^/s which means that InsP_3_ may only diffuse <5μm in 1 s (Dickinson et al., 2016). We have previously presented models of Ca^2+^ oscillations based upon Ca^2+^ induced InsP_3_ formation and InsP_3_ induced opening of Ca^2+^ release channels (Theodoridou et al., 2013). We have now simulated the Ca^2+^ waves in mouse eggs using a similar set of equations in a two-dimensional model of the Ca^2+^ wave. Figure 4 shows that with the source of Ca^2+^ stimulated InsP_3_ production at the periphery (plasma membrane) it is not possible to generate a Ca^2+^ wave through the egg cytoplasm, and only a concentric pattern of Ca^2+^ release occurs. We previously presented evidence for PIP_2_ being present in intracellular vesicles spread throughout the cytoplasm in mouse eggs (Yu et al., 2012). These could provide a source of InsP_3_ that might carry a Ca^2+^ wave through the cytoplasm if they are sufficiently dispersed. In Figure 4 we show simulations based upon Ca^2+^ induced InsP_3_ generation where the PIP_2_ is dispersed on vesicles at different distances apart (from 2 to 4 μm). Our simulations show that when the PIP_2_ vesicles are within 2 or 3 μm of each other a rapid Ca^2+^ can be generated, but that once the PIP_2_ is more than 3 μm the Ca^2+^ increase fails to occur. These results suggests that PIP_2_ needs to be present on vesicles spaced <3 μm apart in the cytoplasm in order to propagate a rapid Ca^2+^ wave of the type seen in fertilizing and PLCζ injected eggs.

**Figure 5:**
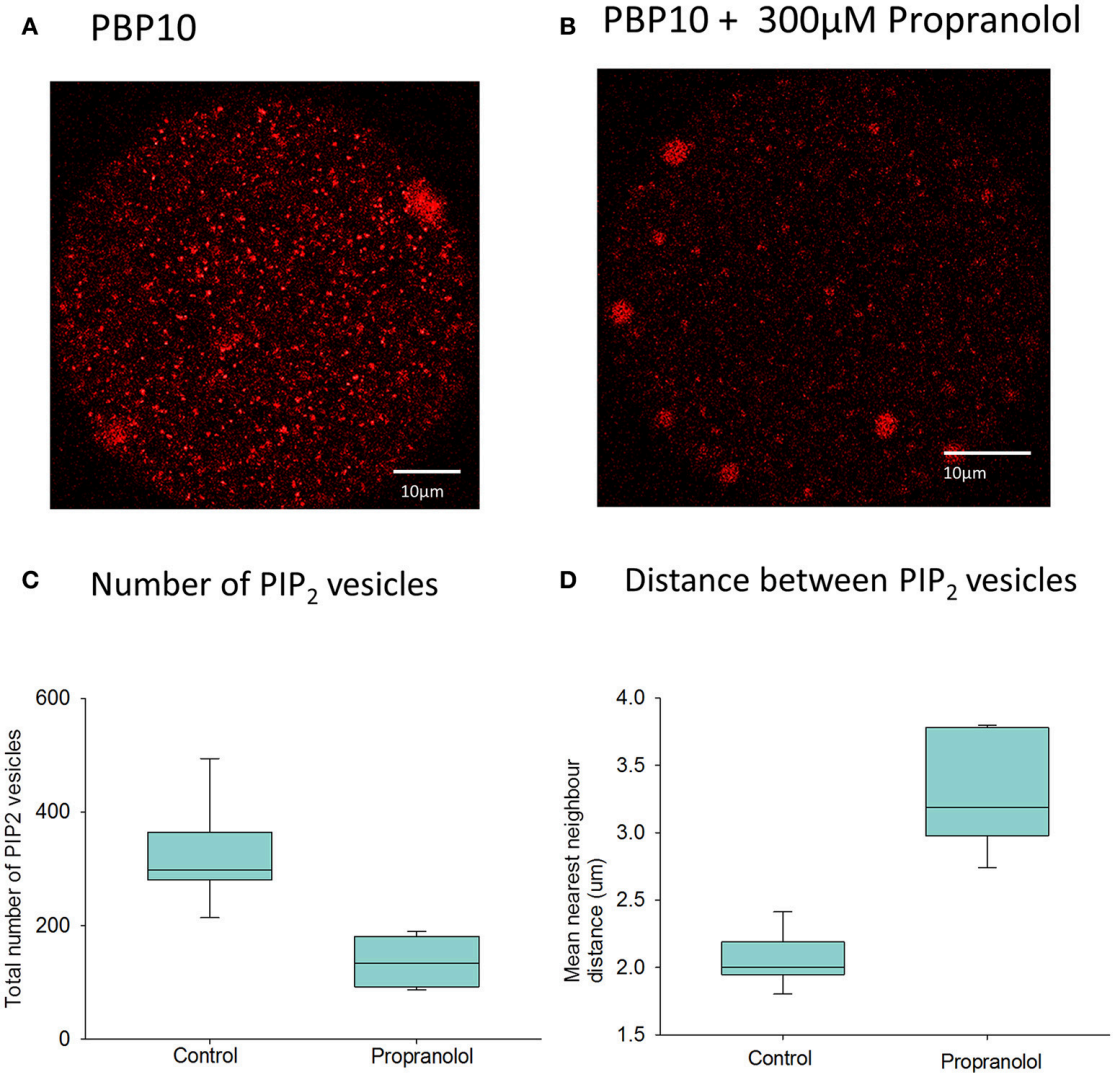
PIP_2_ distribution in mouse eggs using PBP10. In **(A)** an example is shown of the distribution of fluorescence of PBP10 in a mouse egg 1.5 h after injection of PBP10 (*n* = 21). Scale bars are 10 μm. **(A)** nearest neighbor analysis indicated that the mean vesicle distance for all 21 control eggs is 2.2 μm. In **(B)** an example is shown of an egg injected with PBP10 where and incubated in media with 300 μM propranolol (*n* = 13). In **(C)** particle analysis (*n* = 14 eggs) indicates that the mean vesicle diameter is 0.89 μm and the mean number of vesicles present per egg is 298.9. **(C)** Shows a plot of the total number of PIP_2_ positive vesicles present in eggs following injection of PBP10 using particle analysis. Results are shown for both eggs incubated in standard M2 media (control) (mean number of vesicles = 324, *n* = 7) and those incubated in M2 containing 300 μM propranolol during imaging (mean number of vesicles = 131, *n* = 7). There is a significant reduction in the number of PBP10 vesicles following propranolol treatment compared to control media (*p* = < 0.001, Student's *T-test*). **(D)** shows a plot of the mean nearest neighbor distances of PIP_2_ positive vesicles present in eggs. The results are shown for parallel groups of eggs incubated in standard M2 media (control) (mean distance = 2.0 μm, *n* = 7) and for those incubated in M2 containing 300 μM propranolol during imaging, (mean distance = 3.3 μm, *n* = 7). A Mann–Whitney *U-test* showed a significant increase in the distance between the PBP10 vesicles following propranolol treatment compared to control media (*p* = < 0.001).

Previous evidence for the existence of PIP_2_ within the cytoplasm of eggs came from studies using antibodies to PIP_2_ (Yu et al., 2012). Gelsolin is a protein that has been shown to bind to PIP_2_, and contains a short peptide sequence responsible for PIP_2_ binding (Cunningham et al., 2001). We injected mouse eggs with PBP-10, which is a probe in which rhodamine is coupled to a gelsolin peptide that binds PIP_2_. Figure 5A shows a mouse egg injected with PBP-10. After >1 h the fluorescence of PBP-10 could be predominantly seen in many small vesicles throughout the egg cytoplasm, with the occasional larger aggregate. This supports the hypothesis that PIP_2_ is localized in vesicles within mouse eggs (Yu et al., 2012). Further examination of these vesicles using particle analysis indicates that they are distributed throughout the whole egg cytoplasm. Interestingly, following nearest neighbor analysis, we found that these vesicles were ~2 μm apart (Figures 5A,D). This suggests that these PIP_2_ containing vesicles are within the correct distance predicted to produce the rapid rising phase of 1 s for each wave as predicted by our mathematical modeling.

We have previously sought to modify the level of PIP_2_ in mouse eggs using various phosphatases, but without success. Internal membranes in somatic cells do not in general contain much PIP_2_, but one organelle where PIP_2_ and DAG have been reported in some cells is the Golgi apparatus. In mature mammalian eggs, like mitotic cells, the Golgi is fragmented into small vesicles (Moreno et al., 2002; Axelsson and Warren, 2004). It has been shown that propranolol blocks DAG synthesis in Golgi membranes and leads to a loss of Golgi structure (Asp et al., 2009). We applied propranolol to mouse eggs injected with PBP10 and found a marked loss of staining (Figure 5B). Further particle analysis showed that the mean number of these PIP_2_ vesicles was significantly reduced following the addition of propranolol (Figure 5C). Furthermore, the distance of these vesicles from each other was significantly increased in those eggs treated with propranolol (Figure 5D). The overall total fluorescence of the vesicles was seen to reduce by approximately half from a mean of 5.77 × 10^4^ RFU (*n* = 7) in control eggs to a mean of 2.93 × 10^4^ RFU (*n* = 7) in those eggs treated with propranolol. This difference was significant following a Student's *T-test* (*p* = 0.006). This implies that propranolol is affecting PIP_2_ levels in cytoplasmic vesicles.

**Figure 6:**
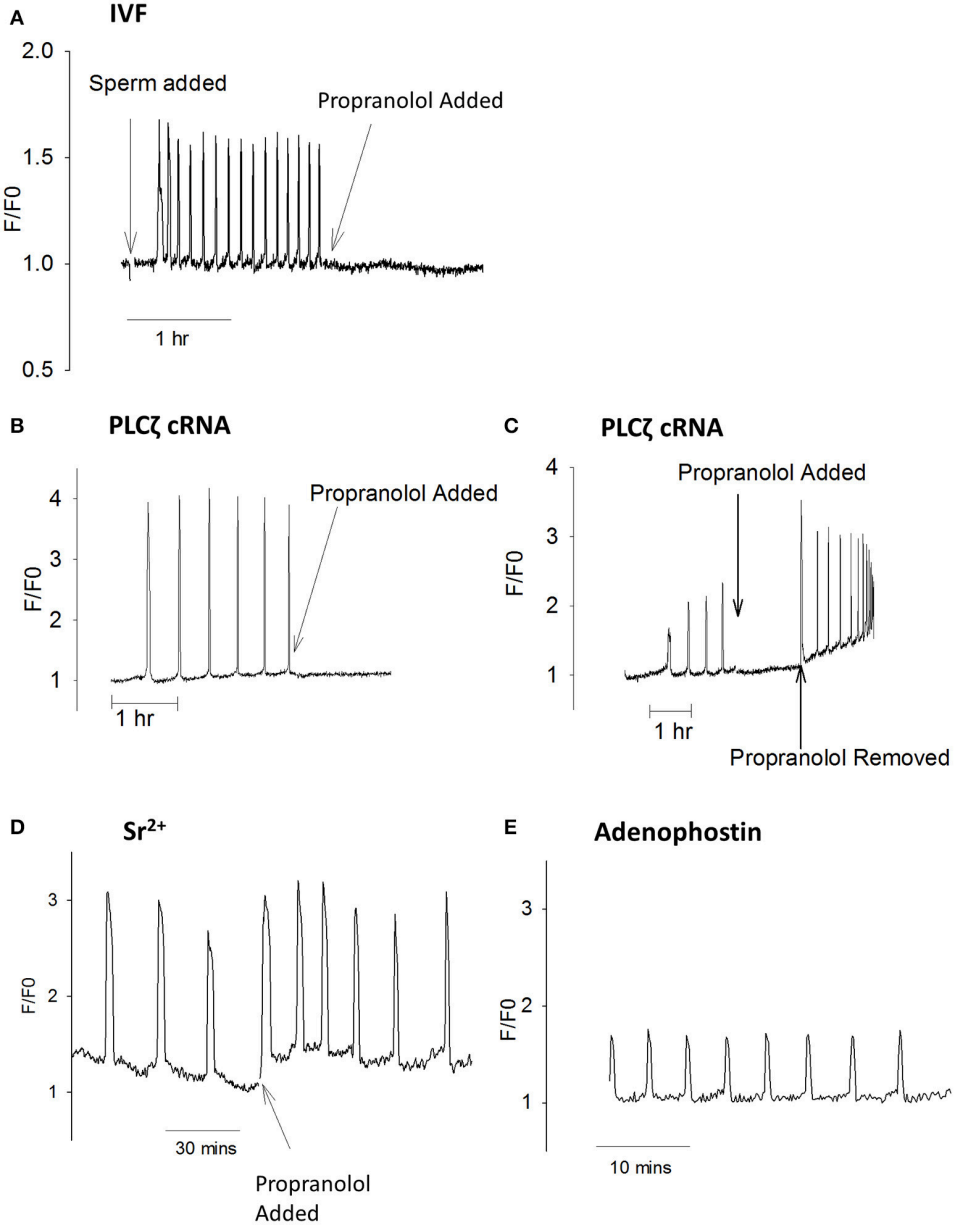
Ca^2+^ oscillations blocked by propranolol. In **(A)** an example is shown of a mouse egg undergoing Ca^2+^ oscillations at fertilization where the addition of 300 μM propranolol inhibited subsequent oscillations (*n* = 13 eggs). Before addition of propranolol the mean frequency was 12.2 ± 1.14 spikes/h with all eggs oscillating. After adding propranolol there were 0.8 ± 0.23 spikes /h (a significant difference from before propranolol, *p* = < 0.0001). 6/13 eggs stopped oscillating immediately, 4/13 eggs had one Ca^2+^ spike, and 3/13 has 2 spikes in an hour. **(B)** shows PLCζ cRNA (pipette concentration = 0.02 μg/μl) induced Ca^2+^ oscillations inhibited by propranolol (*n* = 21 eggs). Before propranolol all eggs oscillated with 4.3 ± 0.46 spikes/h. After addition of propranolol there were 0.95 ± 0.25 spikes/h (a significant difference *p* < 0.0001). With propranolol, 10/21 eggs stopped Ca^2+^ oscillations, 6/21 showed a single spike, and 5/21 had >1 Ca^2+^ spike. In **(C)** an example is shown of an egg where PLCζ induced Ca^2+^ oscillations were blocked by the addition of propranolol but then oscillations were restored when propranolol was washed out (typical of *n* = 8 eggs). Before propranolol, all eggs oscillated with 6.7 ± 1.3 spikes/h. After propranolol this decreased to 1.33 ± 0.29 spikes/h, with all oscillations stopping after 2 spikes. When propranolol was removed there were 10 ± 0.55 spikes in 30 min. Adding propranol and then removing it both caused significant changes in the number of Ca^2+^ spikes (*p* < 0.001). **(D)** shows an example of an egg undergoing Ca^2+^ oscillations in response to Sr^2+^ media where propranolol was subsequently added (*n* = 10 eggs). Before propranolol all eggs oscillated with 4.1 ± 0.29 spikes/h. After adding propranolol all eggs continues to oscillate with 3.9 ± 0.66 Ca^2+^ spikes/h (not significantly different). In **(E)** an example is shown of an egg undergoing Ca^2+^ oscillations in response to microinjection of 5 μM adenophostin in media that contained 300 μM propranolol form the start of the experiment (*n* = 8 eggs). In propranolol there were 5.5 ± 0.51 Ca^2+^ spikes 30 min, compared with 10.4 ± 0.71 spikes/30 min (*n* = 11) for eggs in media with HKSOM. This is significantly different (unpaired *t-test, p* = < 0.0001).

Since proproanolol appears to reduce PIP_2_ inside eggs, we investigated the effect of propranolol on Ca^2+^ oscillations. Figure 6A shows that propranolol addition to eggs undergoing Ca^2+^ oscillations in response to fertilization by IVF were rapidly blocked. Figure 6B shows the same effect of propranolol on those eggs stimulated by PLCζ cRNA. The inhibition by propranolol was associated with a slight decline in Ca^2+^ levels and the inhibition was reversed upon removal of propranolol (Figure 6C). However, whilst it blocked sperm and PLCζ induced responses, propranolol did not block Ca^2+^ oscillations induced in eggs by Sr^2+^ media, or by injection of the IP3R agonist adenophostin (Figures 6D,E). These data show that the inhibitory effects of propranolol are both reversible and specific to PLCζ and sperm induced Ca^2+^ oscillations. They support the proposal that PIP_2_ in vesicles in the cytoplasm of mouse eggs is important for the generation of PLCζ induced Ca^2+^ oscillations.

## Discussion

The Ca^2+^ oscillations seen in mammalian eggs at fertilization have distinct characteristics compared with those seen in somatic cell types (Dupont and Goldbeter, 1994; Politi et al., 2006). The oscillations at fertilization are low frequency, and long lasting, but they have a very rapid rising phase that occurs throughout the whole cytoplasm of a very large cell, in less than a second. Considerable evidence suggests that PLCζ is the primary stimulus for these Ca^2+^ oscillations (Saunders et al., 2002). The current data shows that PLCζ induced Ca^2+^ oscillations are driven by Ca^2+^ induced InsP_3_ formation. In contrast, we show that Sr^2+^ media sensitizes eggs to InsP_3_ induced Ca^2+^ release. Hence, there are at least two different mechanisms for generating Ca^2+^ oscillations in mouse eggs. Our data also implies that the substrate of PLCζ, PIP_2_, needs to be localized in a finely distributed source within the egg in order to generate fast Ca^2+^ wave, and we present evidence that such vesicular PIP_2_ is required for PLCζ induced Ca^2+^ oscillations.

There are two fundamentally different classes of models for InsP_3_ induced Ca^2+^ oscillations in cells. One relies on the properties of InsP_3_ receptor and implies that stimulation involves an elevated but monotonic or constant elevation of InsP_3_ levels. The other involves a positive feedback model of InsP_3_ induced Ca^2+^ release and Ca^2+^ induced InsP_3_ formation. It is possible to determine which one of these two model types applies by studying the Ca^2+^ responses after triggering a large pulsed release of InsP_3_ (Sneyd et al., 2006). The IP3R based models respond to a pulse of InsP_3_ by temporarily increasing the frequency of Ca^2+^ oscillations, whereas the Ca^2+^-induced InsP_3_ formation models show an interruption in the series of Ca^2+^ transients with a resetting of the phase of the oscillations (Sneyd et al., 2006). We previously presented preliminary evidence for an interruption in the series transients with sperm or PLCζ induced Ca^2+^ oscillations responding to a pulse of InsP_3_ (Swann and Yu, 2008). We now show that the response of PLCζ induced Ca^2+^ oscillations to a sudden large pulse of InsP_3_ is clearly characterized by a resetting of the phase of oscillations. This means that InsP_3_ has to be a dynamic variable in the oscillation cycle and that it will undergo oscillations in close phase with the oscillations in Ca^2+^. Small oscillations in InsP_3_ have been recorded previously in response to high frequency Ca^2+^ oscillations achieved with high concentrations of PLCζ (Shirakawa et al., 2006). The sensitivity of such indicators may be limited since we can now assert that InsP_3_ oscillations should occur with all the PLCζ induced Ca^2+^ oscillations and, most significantly, that increased InsP_3_ production plays a causal role in generating each Ca^2+^ rise. We have also shown here that Sr^2+^ works via an entirely different mechanism in mouse eggs. The increase in frequency of Ca^2+^ oscillations caused by uncaging InsP_3_ indicates that Sr^2+^ induced oscillations rely on the properties of the IP3R. This is supported by the finding that Sr^2+^ media sensitized mouse eggs to InsP_3_ pulses, which is consistent with the idea that Sr^2+^ stimulates the opening of InsP_3_ receptor. These data overall show that mouse eggs have more than one mechanism for generating Ca^2+^ oscillations and that in some cases Ca^2+^ oscillations can appear to be similar in form, but be generated by different mechanisms.

It is well-established that Ca^2+^ free media leads to a reduction or abolishment of Ca^2+^ oscillations in response to fertilization or PLCζ injection in mammalian eggs (Igusa and Miyazaki, 1983; Lawrence and Cuthbertson, 1995). It has been assumed that this reflects the loss or some reduction of Ca^2+^ in the endoplasmic reticulum (Kline and Kline, 1992b). However, our data suggest that a reduction in resting, or interspike, cytosolic Ca^2+^ levels also occurs during incubation in Ca^2+^ free media. The reduction in cytosolic Ca^2+^ is apparent with the Ca^2+^ dye we used because it is dextran linked and hence is only within the cytosolic compartment, and because the K_d_ for OGBD and Ca^2+^ is around 250 nm. The reduction in resting Ca^2+^ level appears to cause the inhibition of Ca^2+^ oscillations, rather than a loss of Ca^2+^ store content, because low concentrations of thapsigargin, which will only reduce Ca^2+^ stores content further, actually restores Ca^2+^ oscillations in Ca^2+^ free media. The restoration of such Ca^2+^ oscillations by thapsigargin in our experiments was clearly associated with a rise in the basal Ca^2+^ level. PLCζ induced Ca^2+^ oscillations eventually stopped in Ca^2+^ free media with thapsigargin and this could be because Ca^2+^ stores eventually became depleted. However, the earlier rise in cytosolic Ca^2+^ seems to be a stimulatory factor because low concentrations of thapsigarin, which raise basal Ca^2+^, could also increase the frequency Ca^2+^ oscillations in normal media. This was associated a reduction in the amplitude of Ca^2+^ spikes, presumably because Ca^2+^ store content is reduced. Low concentrations of thapsigargin have also previously been found to stimulate Ca^2+^ oscillations in immature mouse oocytes (Igusa and Miyazaki, 1983; Lawrence and Cuthbertson, 1995). Hence, these data together imply that cytosolic Ca^2+^ level, rather than Ca^2+^ store content is the more significant factor setting the frequency and occurrence of physiological Ca^2+^ oscillations.

These data are consistent with recent studies measuring free Ca^2+^ inside the endoplasmic reticulum in mouse eggs (Wakai et al., 2013). It was shown that a reduction in ER Ca^2+^ occurs following each Ca^2+^ spike, but that there is no correlation between when a Ca^2+^ transient is initiated and the level of Ca^2+^ in the ER (Wakai et al., 2013). Whilst it is obvious that some Ca^2+^ store refilling will occur in the intervals between Ca^2+^ spikes, it is not likely that this sets of the pace of the low frequency Ca^2+^ oscillations characteristic of mammalian eggs. We suggest that the pacemaker that determines when the next Ca^2+^ transient occurs after PLCζ injection is more likely to be the rise in cytosolic Ca^2+^. A gradual rise in cytosolic Ca^2+^ between spikes is evident in the PLCζ induced Ca^2+^ oscillations in all the traces in this paper. This gradual Ca^2+^ increase could promote a gradual rise in InsP_3_ that will eventually lead to a positive feedback loop and a regenerative Ca^2+^ wave.

Although the Ca^2+^ oscillations triggered by fertilization in mammalian eggs are of low frequency, each of the waves of Ca^2+^ release that causes the upstroke of a Ca^2+^ increase crosses the egg remarkably quickly. Previous analysis of the wave dynamics of Ca^2+^ release in mammalian eggs have suggested that the rising phase of each Ca^2+^ oscillation is ~1 s. This correlates with the speed of the Ca^2+^ wave that crosses the egg at a speed of >50 μm/s. This is significant because the diffusion coefficient of InsP_3_ in intact cells has been estimated to be no more that 10 μm^2^/s (Dickinson et al., 2016). In models where InsP_3_ is elevated at a constant level during Ca^2+^ oscillations the restricted diffusion of InsP_3_ is not an issue because it will reach a steady state concentration across the egg. However, our data shows that Ca^2+^ and InsP_3_ act together in a positive feedback loop to cause each propagating Ca^2+^ wave. In this case the diffusion of InsP_3_ could be a rate limiting step. If all the InsP_3_ is generated in the plasma membrane then our simulations show that a Ca^2+^ induced InsP_3_ production model cannot generate Ca^2+^ waves through the egg cytoplasm. If we simulate the InsP_3_ production from discrete sites within the egg cytoplasm then rapid Ca^2+^ waves of some type can be generated, but full waves can only be seen when the sites of InsP_3_ generation are within 3 μm of each other. This suggests that in order to explain both the fast Ca^2+^ waves and the basic mechanism of sperm or PLCζ induced oscillations in mammalian eggs, the PIP_2_ substrate has to be dispersed in sites throughout the egg cytoplasm. This conclusion is similar to that previously suggested for ascidian oocyte at fertilization which also show rapid Ca^2+^ waves and oscillations (Dupont and Dumollard, 2004).

We previously reported evidence for a vesicular source of PIP_2_ in mouse eggs using immunostaining (Yu et al., 2012). The vesicular staining with PIP_2_ antibodies closely mimics the distribution of PLCζ also probed with antibodies (Yu et al., 2012). We now report a similar pattern of vesicular staining using another probe (PBP10) which based upon the PIP_2_ binding region of gelsolin (Cunningham et al., 2001). This probe has the advantage that it is microinjected into eggs that can then be imaged whilst still alive and so does not require the fixation and permeabilization procedures associated with immunostaining. It gives a very different pattern of staining from another commonly used probe for PIP_2_ which is the GFP-PH domain which localizes predominantly to the plasma membrane in mouse eggs (Halet et al., 2002). However, the PH domain of PLCδ1 that is used for the localization of PIP_2_ in such a probe may also bind cholesterol so may be influenced by factors other than PIP_2_ (Rissanen et al., 2017). It is entirely possible that PBP10 is also influenced by factors other than PIP_2_, but it is noteworthy that the PBP10 staining gives a vesicular localization pattern that closely resembles that seen with the PIP_2_ antibodies. The fact that two very different methods for localization PIP_2_ in eggs, immunostaining with a monoclonal antibody and a fluorescently tagged peptide, show such a distinctive and similar pattern of localization provides good evidence that PIP_2_ is indeed localized within vesicles in the cytoplasm in of mouse eggs. Using the live cell probe, PBP10, we were able to estimate that the apparently PIP_2_ containing vesicles we see in eggs are within about 2 μm of each other. This distance closely correlates with the estimate of how close PIP2 vesicles need to be in order to propagate a Ca^2+^ wave across the egg within ~1 s. Hence, our data provide a coherent view of PLCζ induced Ca^2+^ release in eggs in which Ca^2+^ induced InsP_3_ formation from closely spaced vesicles containing PIP_2_ accounts for the upstroke of each Ca^2+^ rise.

The precise nature of the PIP_2_ containing vesicles that appear to exist in mouse eggs is unclear. We have tested a number of antibodies and other probes for specific organelles in eggs and found that many either localize to the endoplasmic reticulum or else show only a limited overlap in staining with the PIP_2_ or PLCζ positive vesicles. The identification of PBP10 positive vesicles is further complicated by our finding that its pattern of localization does not persist after fixation and membrane permeabilization (Sanders and Swann, unpublished). In somatic cells, non-plasma membrane PIP_2_ has been found in the Golgi apparatus (De Matteis et al., 2005). Mature mouse egg are unusual compared with somatic cells in that they are arrested in meiosis, which is similar to the mitotic phase of the cell cycle. During mitosis the Golgi fragments to form small vesicles known as the Golgi haze (Axelsson and Warren, 2004), and the Golgi in mouse eggs has been shown to be fragmented into small vesicles (Moreno et al., 2002). The structure of the Golgi and its associated vesicles is maintained by the presence of diacyglycerol (DAG) (Asp et al., 2009). The drug propranolol disrupts Golgi resident proteins and lipids by inhibiting DAG production and as a result, it also disrupts Golgi-ER trafficking (Asp et al., 2009). Interestingly propranolol was found to block Ca^2+^ oscillations triggered by PLCζ and fertilization. This effect was specific in that the same concentration of propranolol did not effect oscillations when added to other Ca^2+^ releasing agents such as Sr^2+^ media which causes a pattern of oscillations most similar to fertilization. The small effect on adenosphostin induced Ca^2+^ oscillations is unlikely to be sufficient to explain the effects of propranolol because it was only a 2-fold decrease in oscillations compared the cessation of oscillations after propranolol in most eggs that were fertilized or injected with PLCζ. It is also noteworthy that the Ca^2+^ levels remained low in propranolol treated eggs, and that its effects were reversible. In mouse eggs we found that propranolol also decreased the number of the PIP_2_ containing vesicles and the mean distance between vesicles, therefore presumably, the availability of the vesicular PIP_2_ to propagate a Ca^2+^ wave. This effect could be because propranolol disrupts the structure of the vesicles or because trafficking between the Golgi and the ER is inhibited. Whatever the actual mechanism, the loss of PIP_2_ after treatment with propranolol supports our hypothesis that these vesicles are required for generating Ca^2+^ oscillations in eggs in response to sperm or PLCζ. Since there is evidence for intracellular PIP_2_ on organelles in frog and sea urchin eggs, which also show Ca^2+^ waves at fertilization, it is attractive to speculate that intracellular PIP_2_ is an important feature that allows eggs to generate the Ca^2+^ signal needed for egg activation.

## Author contributions

JS: Performed some of the Ca^2+^ measurements and the PIP_2_ imaging experiments, analyzed data, and co-wrote the manuscript; BA and AM: Performed Ca^2+^ measurement experiments on eggs and analyzed data; TW: Produced and analyzed the mathematical simulation; KS: Conceived the study, directed experiments and co-wrote the manuscript. All authors approved the final manuscript.

### Conflict of interest statement

The authors declare that the research was conducted in the absence of any commercial or financial relationships that could be construed as a potential conflict of interest.
